# Supplementary material for: A mesoscale phase-field model of intergranular liquid lithium corrosion of ferritic/martensitic steels
Source: Npj Mater Degrad. 2025 Jun 10;9(1):68. doi: 10.1038/s41529-025-00616-4 (PMC12151869; doi:10.1038/s41529-025-00616-4)
Supplement: Supplementary file 1 — Supplementary Information [file 41529_2025_616_MOESM1_ESM.pdf]

Supplementary Information

Ten different microstructures with an average grain size of 20 μm and various GBs at the exposed surface considered in this work are given in Figs. S.1, S.2, and S.3. Representative microstructures with an average grain size of 10 μm and 10 GBs at the exposed surface are depicted in Fig. S.4. Representative microstructures with an average grain size of 40 μm and 2 GBs at the exposed surface are shown in Fig. S.5.

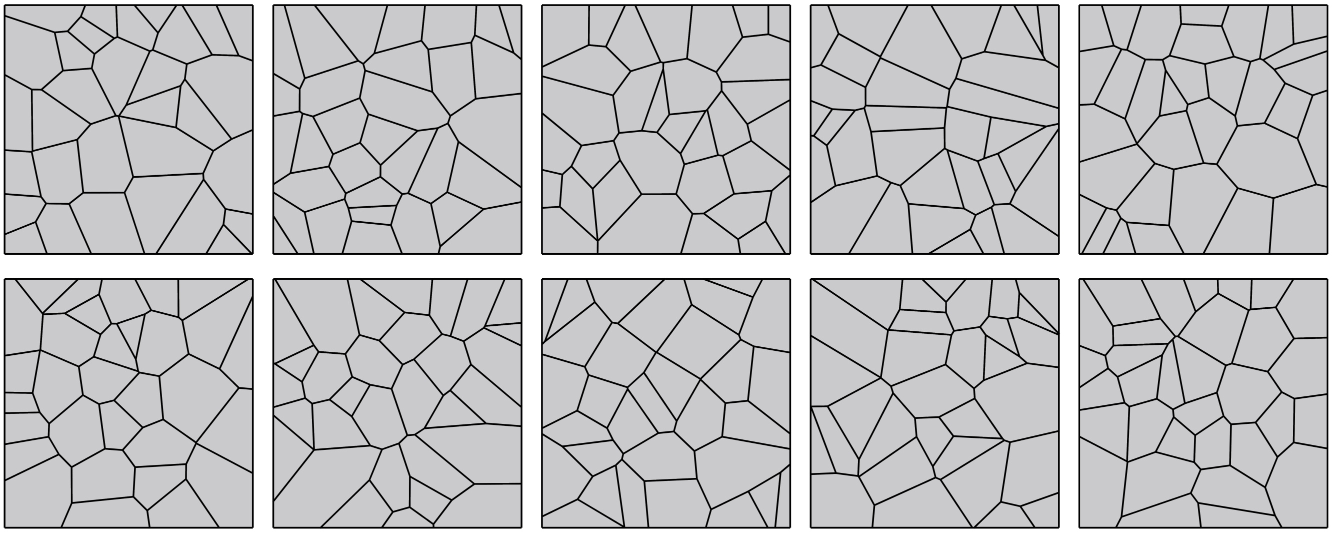

Figure S.1: Ten microstructures with an average grain size of 20 μm and 6 GBs at the exposed surface.

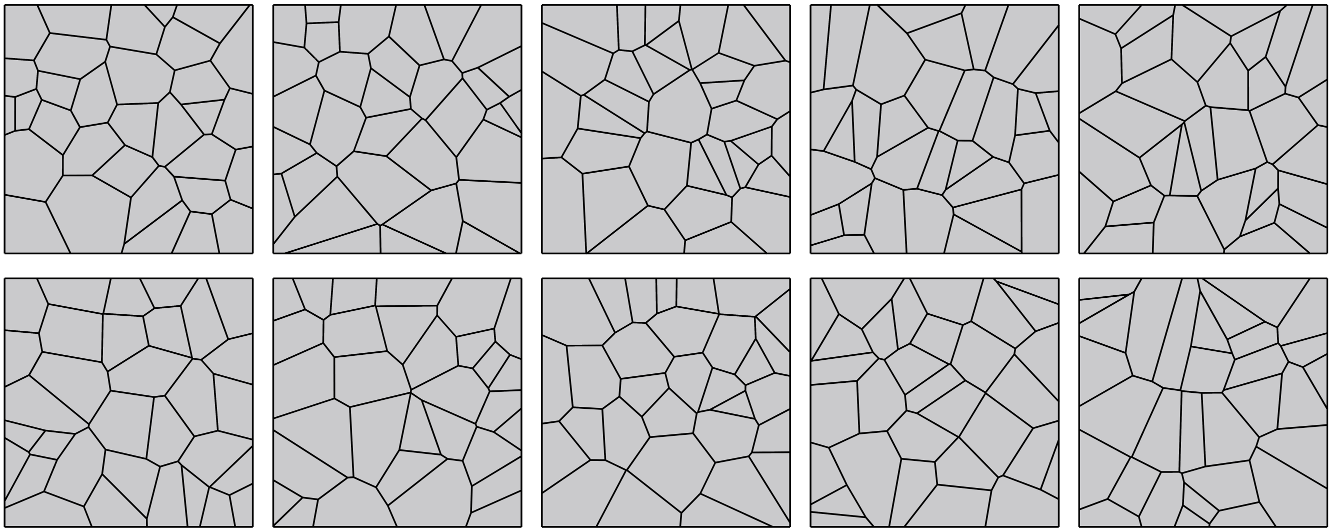

Figure S.2: Ten microstructures with an average grain size of 20 μm and 5 GBs at the exposed surface.

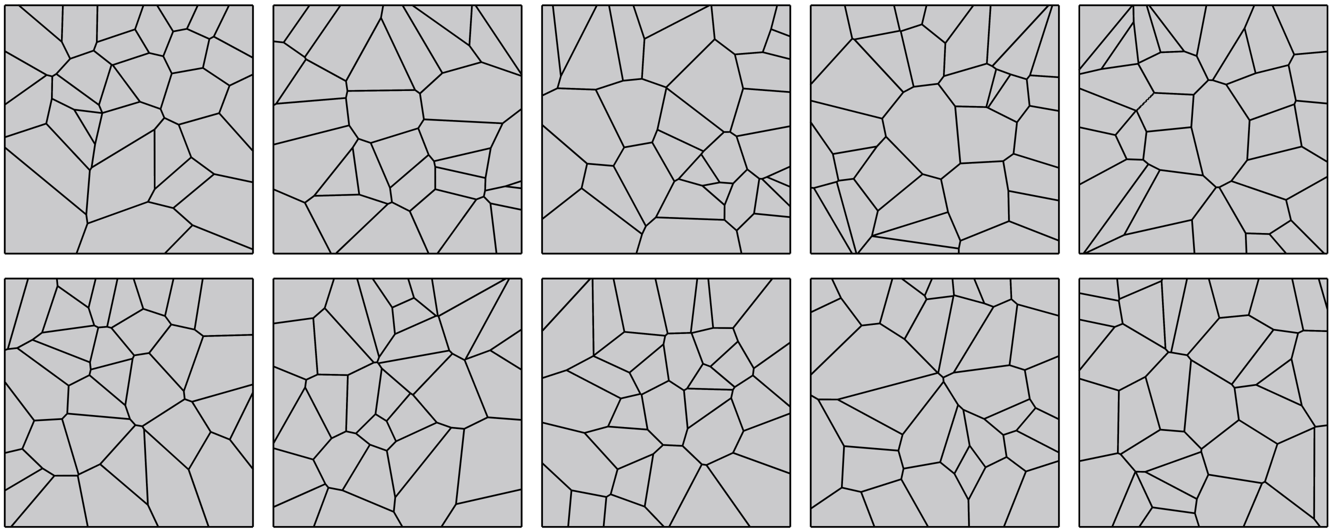

Figure S.3: Ten microstructures with an average grain size of 20 μm and 7 GBs at the exposed surface.

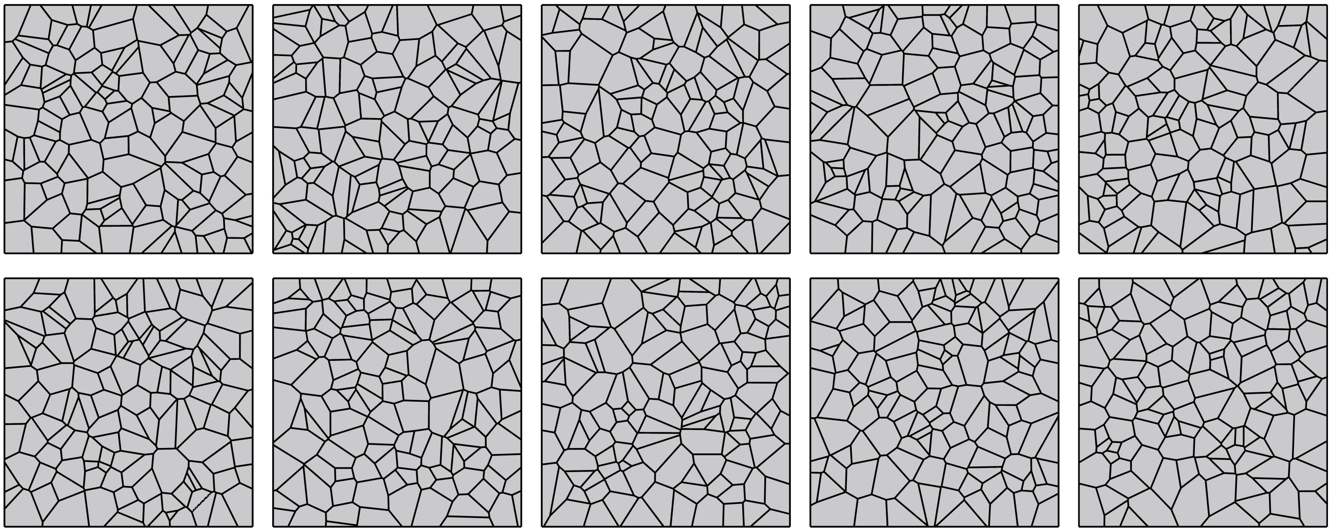

Figure S.4: Ten microstructures with an average grain size of 10 μm and 10 GBs at the exposed surface.

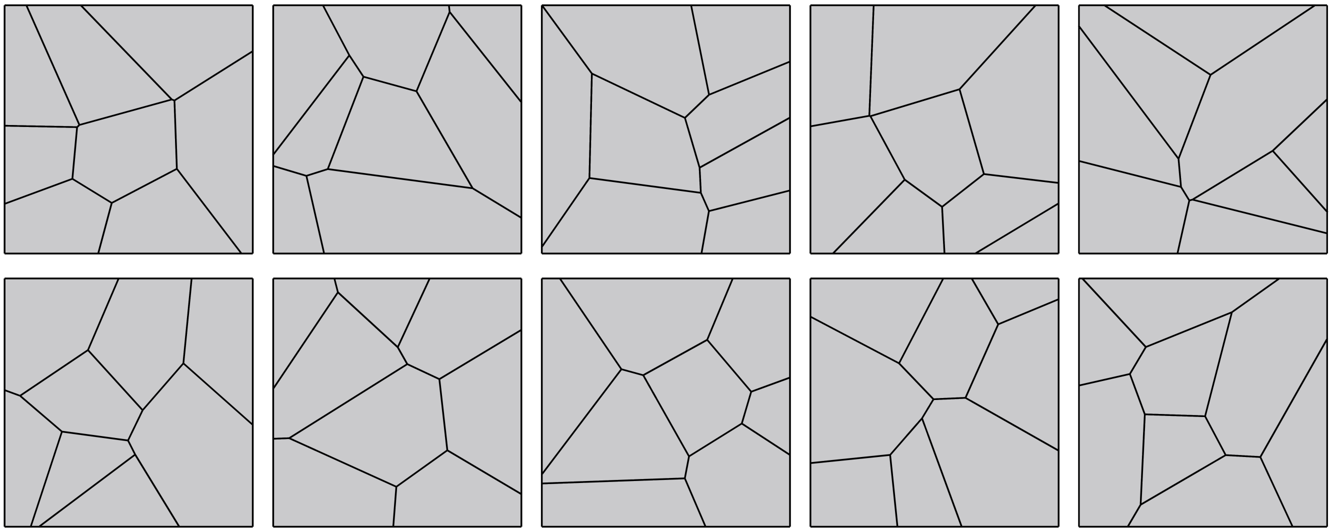

Figure S.5: Ten microstructures with an average grain size of 40 µm and 2 GBs at the exposed surface.
